# Combination of Oligofructose and Metformin Alters the Gut Microbiota and Improves Metabolic Profiles, Contributing to the Potentiated Therapeutic Effects on Diet-Induced Obese Animals

**DOI:** 10.3389/fendo.2019.00939

**Published:** 2020-02-25

**Authors:** Qingzhong Li, Rui He, Fengmei Zhang, Jian Zhang, Shihai Lian, Hongxia Liu

**Affiliations:** ^1^Department of Clinical Pharmacy, School of Pharmacy, Binzhou Medical University, Yantai, China; ^2^Shandong First Medical University & Shandong Academy of Medical Sciences, Taian, China; ^3^Department of Rehabilitation Medicine, Affiliated Hospital of Binzhou Medical University, Binzhou, China; ^4^Second Department of Endocrinology, Taian City Central Hospital, Taian, China; ^5^Department of Thoracic Surgery, Zaozhuang Municipal Hospital, Zaozhuang, China

**Keywords:** metformin, oligofructose, gut microbiota, obesity, T2DM

## Abstract

Accumulating studies implicate that the metformin (MET)- and oligofructose (OFS)-altered gut microbiota may play roles in the improvement of type 2 diabetes mellitus (T2DM) and obesity. However, whether the combined administration of OFS and MET could effectively affect the gut microbiota and improve metabolic profiles remains unknown. Here, we randomized diet-induced obesity (DIO) rats to OFS, MET, or MET+OFS for 8 weeks and demonstrated that the combined administration of OFS+MET possessed potentiated effects on the glycemia, body weight, and gut microbiome. In addition, fecal samples from the MET and MET+OFS group were exchanged and transferred to germ-free rats induced by antibiotics. Not surprisingly, the glucose tolerance and serum levels of endotoxin, free fatty acids (FFA), tumor necrosis factor-α (TNF-α), interleukin-2 (IL-2), and interleukin-6 (IL-6) were all sustainably improved among OFS+MET fecal microbiota-treated DIO rats while the MET fecal microbiota-treated ones presented a relatively reverse trend. Furthermore, transfer of fecal samples from the rats after 8 weeks of treatment to antibiotics-treated germ-free mice significantly improved metabolic profiles, including glucose tolerance and weight reduction in mice that received MET+OFS-altered microbiota. In conclusion, the present study illustrated that the effects of OFS and MET combined treatment on gut microbiota, especially for the MET-induced side effect-related ones, and host metabolism were of greater magnitude than individual OFS or MET treatment in obese rats and mice. Therefore, it is likely that combined administration of OFS and MET may offer a novel and promising strategy for reducing side effects induced by MET and improving metabolic outcomes, particularly glycemia control and weight reduction.

## Introduction

T2DM and obesity are both representative chronic systemic disorders of overweight and hyperglycemia mainly resulting from the genetic and environmental risk factors, including the relative lack of insulin caused by insulin resistance, high-fat and high-sugar diet, and lack of exercise (1–4). Obesity is one of the serious causes of insulin resistance and T2DM as well (5, 6). In recent years, metformin (MET), an oral blood glucose-lowering non-metabolizable compound, becomes the mainstay therapy for T2DM patients, although its mechanism of action remained unclear (7, 8). Administration of MET obviously improves the blood glucose levels and insulin resistance of individuals with T2DM by the inhibition of hepatic gluconeogenesis and opposing the action of glucagon (9). In addition to the hypoglycemic action, MET has gained attention for its pleiotropic effects, especially on targeting metabolic differences between normal and abnormal metabolic signaling, including the positive influence on body weight reduction, lipid profiles, multiple cardiovascular risk, and inflammatory markers, which helped us to develop novel medical applications, such as in obesity (10–12). However, at least 30% of T2DM patients report adverse effects including diarrhea, nausea, vomiting, and bloating, with underlying mechanisms poorly understood. These side effects severely limit the wide application prospects of MET (13–15).

Most recently, attention on the role of the gut microbiota in the development and maintenance of obesity and T2DM is growing rapidly. Changes in insulin sensitivity, energy balance, lipid metabolism, and secretion of gut hormone through altered gut microbiota have all been considered as probable mechanisms (16–20). Not only that, several publications implicate that MET interacts with different gut microbiota and provides support for the notion that altered gut microbiota mediates some of MET's antidiabetic and weight loss effects and also includes the side effects (21–23). Oligofructose (OFS) is a prebiotic that can effectively reduce energy intake and fat mass in individuals with T2DM and obesity. Recent evidence demonstrated that OFS supplementation promotes weight loss and improves glucoregulation in obese and diabetic models via the changes in gut satiety hormones and microbiota (24–26). Studies in animals and humans both suggest that some beneficial effects of MET and OFS on glucose metabolism and weight loss may be microbially mediated. However, whether the combination of OFS and MET has synergetic anti-obesogenic, anti-diabetic, or other metabolism-improving properties currently remains unknown.

The aim of the present study was to examine the combined effects of MET and OFS on gut microbiota and metabolic disorders, especially for obesity and T2DM, in an animal model. Here, we investigate how the combination of OFS (10% wt/wt) and MET (150 mg/kg) affects the metabolism of animals by using rats with diet-induced obesity for 8 consecutive weeks, and the glucose tolerance, metabolic parameters, and blood biochemistry indexes were carefully evaluated. After the treatment of antibiotics, the DIO rats treated with MET and MET+OFS were both divided into two groups receiving fecal microbiota from the two groups to investigate whether combination of MET and OFS could effectively reduce the side effect-related indexes and improved metabolic disorders. Further, we also transferred the fecal microbiota from DIO rats treated with OFS, MET, and OFS+MET, respectively, to antibiotics-induced germ-free mice so as to evaluate the effects of MET-altered microbiota on host glucose metabolism.

## Materials and Methods

### Animals and Treatments

The Animal Care Committee of Binzhou Medical University approved the protocol of animal experiments, with approval codes BAC18-115 and BAC19-056. Male Wistar rats and C57BL/6J mice were purchased from Shandong Laboratory Animal Center (Shandong, China) and housed on a 12-h light–dark cycle, at a temperature of 24°C. Male DIO rats and mice were fed a diabetogenic diet, which is high-fat diet with 60% kcal from fat [high-fat diet (60%) diet D12492], for a minimum of 4 months, and the final experimental animals used were between the ages of 7 and 8 months. Wistar rats were randomly assigned to one of four groups: (1) Saline; (2) 10% (wt/wt) OFS (Solarbio, Beijing, China); (3) MET (Sigma, 150 mg/kg); or (4) 10% OFS+MET, for 6 weeks. The dose of OFS and MET was selected based on previous reports (24, 27). ATM Skirrow (including 5 mg/ml neomycin, 5 mg/ml bacitracin, and 2 mg/ml natamycin) was brought from TOKU-E (USA). Food intake and body weight were measured daily. One day prior to sacrifice, rats were lightly anesthetized with isoflurane and body composition was measured via DXA scan with software for small animals. Using blood samples collected from DIO rats, blood biochemical indexes including HbA1c were measured with an automatic biochemical analyzer according to the manufacturer's instructions.

### Oral Glucose Tolerance Test

After overnight fasting (12 h), 2 g/kg glucose was orally administered and blood was collected at 0, 15, 30, 60, 90, and 120 min. Blood glucose was measured immediately with a OneTouch blood glucose meter (Johnson & Johnson, America). The insulin level was detected by the rat insulin ELISA kit (Millipore, Germany).

### Detection of Plasma Satiety Hormones and Other Indexes

Plasma amylin, ghrelin, PYY, CCK, glucose-dependent insulinotropic polypeptide (GIP), and plasma inflammation-associated indexes, including endotoxin, TNF-α, IL-2, and IL-6, were measured using rat ELISA kits (Millipore, Germany). In mice, the levels of leptin, resistin, non-esterified fatty acid (NEFA), tumor necrosis factor-α (TNF-α), adiponectin, and IL-6 were also detected using ELISA kits purchased from Millipore.

### 16S rRNA Analysis of Fecal Samples

Stool samples from DIO rats were collected after 8 weeks of treatment, quickly frozen using liquid nitrogen, and stored in a freezer (−80°C). The analysis of 16S rRNA sequence was conducted at BGI Genomics (Shenzhen, China). The extraction and quality of microbial genome DNA were conducted using the conventional method of TACB/SDS and measured on agarose gel (1%). V3–V4 region 16S rRNA genes were amplified using previous reported primers and methods (28). Libraries were constructed using the Ion Fragment Library Kit (Thermo, America) according to the manufacturer's protocols and sequenced on an Illumina Miseq PE300 system with a generation of single-end 400-bp reads. All sequences were classified using the NCBI BLAST and SILVA databases. Distance calculation, operational taxonomic units (OTU) cluster, rarefaction analysis, and estimator calculation (α-diversity and β-diversity) were performed by qiime2.

### Transplantation of Fecal Microbiota

Fecal samples were obtained and then stored immediately after defecation at −80°C for further transplantation. One gram of fecal sample was diluted in 10 ml of PBS and then suspended to obtain supernatant including fecal microbiota. Transfer of fecal microbiota was conducted on both DIO rats and mice treated with antibiotics. As shown in **Figure 6**, from day −56 to −1, DIO rats were randomly assigned to the four groups. At day 0, OGTT and other metabolic profile baseline of rats were measured. Then, the rats were conducted with gavage of antibiotic solution during days 0–7. Further, intragastric administration of 0.2 ml of fecal material was conducted from day 7 to day 21 (14 consecutive days) to pseudo-germ-free rats. The OGTT and hematological index detection was carefully performed at days 22 and 23, respectively. As shown in **Figure 8A**, after a 7-day acclimatization, the pseudo-germ-free mice model was successfully established by administrating a large dose of antibiotic for 10 consecutive days. In the next 14 days (day 1 to day 14), 0.2 ml of the obtained fecal microbiota from the rats was orally transplanted to pseudo-germ-free mice and OGTT tests were subsequently measured.

### Statistical Analysis

All data are presented as the mean ± SEM. The comparison between three treatment groups to the control and normal groups were performed by one-way ANOVA test. *P* < 0.05 was considered significant.

## Results

### Combination of OFS and MET Alters Metabolic Parameters in DIO Rats

**Figure 1:**
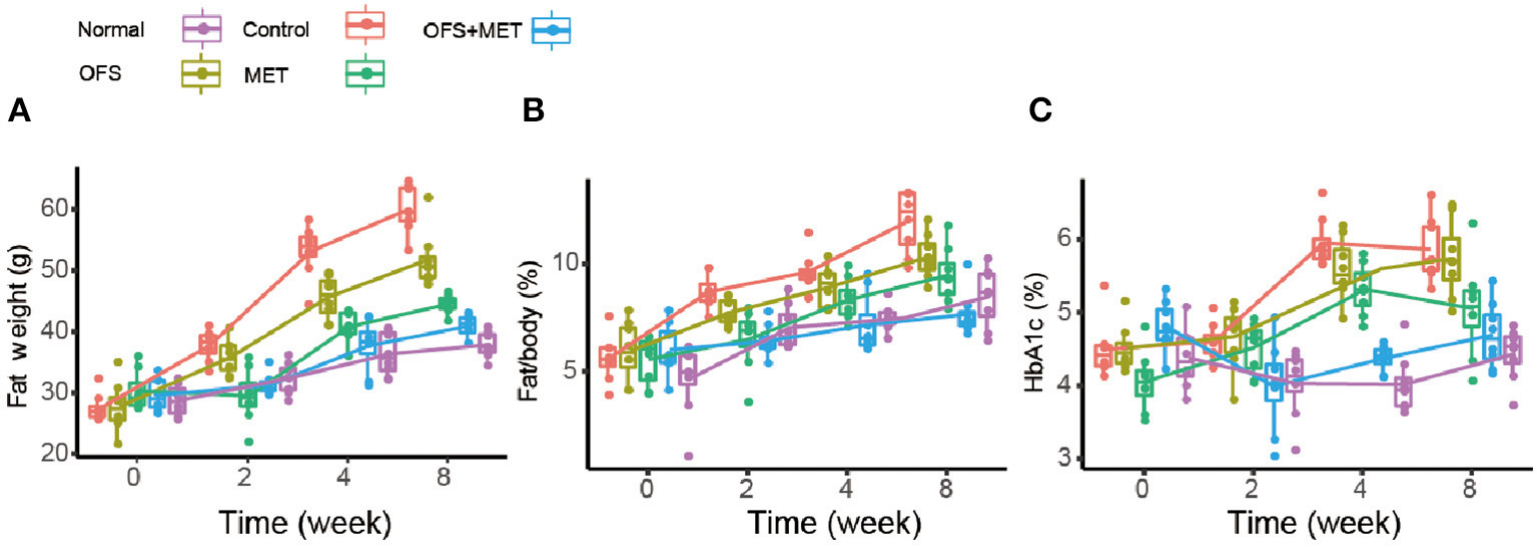
Boxplots (with median) showing **(A)** fat weight, **(B)** %fat/body, and **(C)** %HbA1c before treatment (0 week) and after 2, 4, and 8 weeks in DIO rats randomized to control (C2, C4, and C8), OFS (O2, O4, and O8), MET (M2, M4, and M8; *n* = 8), and OFS+MET (OM2, OM4, and OM8). *P* < 0.05, 0.01, 0.001 using one-way ANOVA vs. saline-treated control (*, **, ***). All data are expressed as mean ± S.E.M. (*n* = 8).

In order to investigate if the combination of OFS and MET affects the metabolism of animals, we treated the DIO rats with placebo, 150 mg/kg of MET, 10% (wt/wt) OFS, and 10% (wt/wt) OFS+150 mg/kg of MET, respectively, for 8 consecutive weeks, using the healthy rats treated with saline as normal group. As a result, the effects of OFS, MET, and OFS+MET treatment on metabolic parameters in DIO rats are shown in Table 1. Compared to the control group, weight gain and final body weight of DIO rats were significantly decreased in both MET (*P* < 0.05) and OFS+MET (*P* < 0.01) groups; however, the effect was not significant, though the result had the same situation in the OFS group. Both MET- and MET+OFS-treated groups showed decrease in food intake compared to the control and OFS-only group. After the 8-week period of the experiment, significant differences in fat weight and fat/body (%) were observed in OFS+MET (40.1 ± 7.0 g, 8.3 ± 1.5%, *P* < 0.02) compared to the control (59.1 ± 9.1 g, 11.9 ± 1.8%) and similar to the normal ones (Figures 1A,B). Administration of both MET and OFS+MET significantly lowers the %HbA1c levels (Figure 1C). The other blood biochemical indexes were also carefully detected at the end of the experiment. As a result, all the sample-treated groups showed a down trending for the levels of triglyceride, cholesterol, and LDL-cholesterol in DIO rats compared to the control. HDL-cholesterol level was also determined and a significant difference was observed only in the OFS+MET-treated group, which was not far from normal values (Table 1).

**Table 1:** Effects of OFS, MET, and OFS+MET on metabolic parameters in DIO rats.

**Parameter**	**Control**	**Normal**	**OFS**	**MET**	**OFS+MET**
Weight gain (g)	56.5 ± 4.4	31.1 ± 4.1	51.7 ± 6.6	45.3 ± 3.0^*^	42.8 ± 3.1^**^
Final weight (g)	495.2 ± 19.1	373.1 ± 12.1	489.5 ± 11.8	421.8 ± 15.4^*^	408.2 ± 12.9^**^
Food intake (g/day)	25.4 ± 3.3	23.1 ± 1.2	25.2 ± 3.1	23.9 ± 2.4	22.1 ± 1.7^*^
Energy intake (kJ/rat/day)	276.1 ± 22.7	240.2 ± 32.3	259.1 ± 29.5	250.9 ± 19.1	248.6 ± 16.4^*^
Triglyceride (mg/ml)	95.1 ± 8.3	81.3 ± 3.2	89.1 ± 8.2	92.2 ± 7.1	90.5 ± 7.0
Cholesterol (mg/ml)	215.2 ± 11.2	153.3 ± 9.3	195.1 ± 11.2	175.2 ± 8.8^*^	170.6 ± 7.3^*^
HDL-cholesterol (mg/ml)	70.9 ± 4.4	95.2 ± 5.8	71.3 ± 6.1	78.3 ± 6.8	87.9 ± 2.8^*^
LDL-cholesterol (mg/ml)	110.1 ± 8.4	78.3 ± 6.3	106.4 ± 7.3	95.2 ± 4.7^**^	94.1 ± 5.9^**^

Values represent the mean ± S.E.M. (n = 8).

* P < 0.05,

** P < 0.01,

*** *P < 0.001 compared to saline-treated control*.

### Effects of OFS, MET, and OFS+MET on Glycemic, Insulinotropic Response, and Insulin Resistance Index in DIO Rats

The effects of OFS and MET treatment on fasting blood glucose, insulin levels, and HOMA value for insulin resistance (IR) in DIO rats are presented in Table 2. Except for the normal group, all the other groups showed significantly increased fasting blood glucose levels and blood insulin level, while the smallest increase was shown in the OFS+MET-treated group. Furthermore, index of insulin resistance was calculated using the obtained concentration of BGL and insulin level accordingly. Not surprisingly, significantly decreased HOMA-IR indices were similarly shown in all treatment groups compared with the control, especially for the OFS+MET-treated group showing the lowest HOMA-IR value and close to the normal.

**Table 2:** Effects of OFS, MET, and OFS+MET on fasting blood glucose, insulin levels and HOMA-IR values.

**Group**	**Blood glucose (mM)**		**Insulin level (μU/ml)**		**HOMA-IR**	
	**0 weeks**	**8 weeks**	**0 weeks**	**8 weeks**	**0 weeks**	**8 weeks**
Saline	4.6 ± 0.1	10.6 ± 0.7	62.8 ± 6.2	391.8 ± 33.1	25.2 ± 1.1	169.2 ± 17.1
Normal	3.2 ± 0.2	3.7 ± 0.2	47.4 ± 3.8	79.2 ± 5.2	10.2 ± 1.2	17.2 ± 2.1
OFS	4.4 ± 0.3	7.4 ± 1.2***	72.2 ± 13.2	227.9 ± 39.3***	26.7 ± 2.4	111.3 ± 19.4**
MET	5.1 ± 0.3	6.9 ± 1.1***	66.2 ± 7.3	190.9 ± 17.3***	28.1 ± 1.4	65.6 ± 2.9***
OFS+MET	4.8 ± 0.4	6.6 ± 0.8***	76.4 ± 6.4	159.4 ± 21.9***	20.1 ± 3.0	25.1 ± 2.0***

P < 0.05, 0.01, 0.001 using one-way ANOVA vs. saline control group (*, **, ***). All data are expressed as mean ± S.E.M. (n = 8).

### Effects of OFS, MET, and OFS+MET on Plasma Levels of Insulin Level and Appetite-Related Hormones During the OGTT in DIO Rats

**Figure 2:**
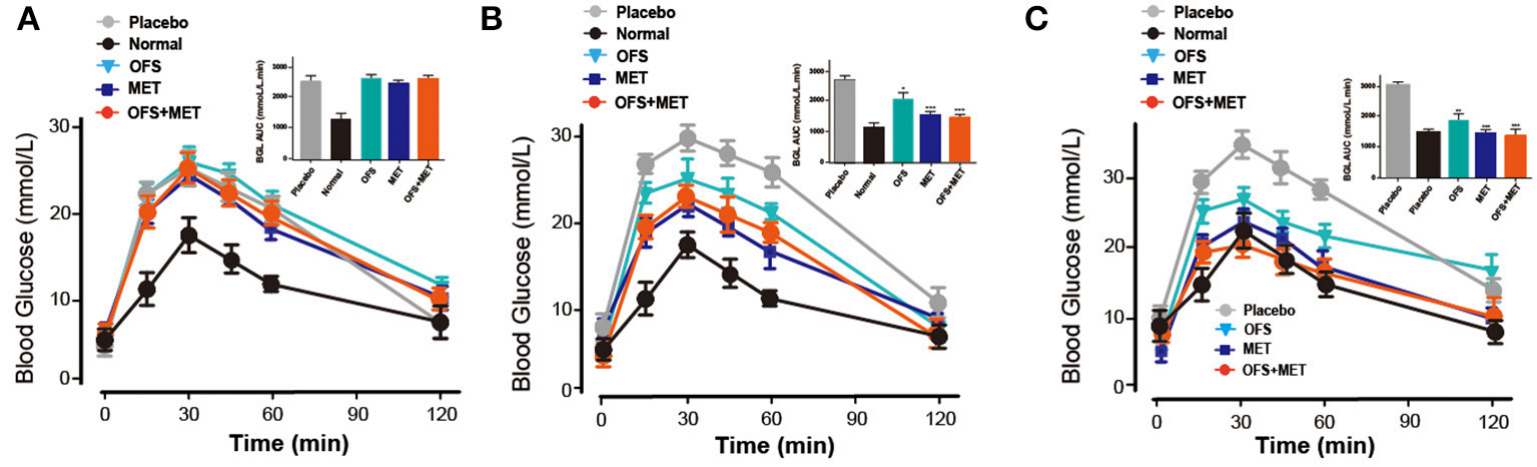
Effects of OFS, MET, and OFS+MET on glucose tolerance of DIO rats. OGTT was conducted at **(A)** Week 0, **(B)** Week 4, **(C)** Week 8. *P* < 0.05, 0.01, 0.001 using one-way ANOVA vs. placebo group (*, **, ***). All data are expressed as mean ± S.E.M. (*n* = 8).

To evaluate whether an 8-week treatment of OFS, MET, and OFS+MET helped to improve the glucose metabolism in DIO rat, OGTT tests were conducted before and during the chronic treatment (week 0, week 4, and week 8). As is presented in Figure 2, all of the MET, OFS, and MET+OFS treatment exert an obvious improvement on the glucose tolerance of the DIO rats, which were closer to the normal group compared with the control. DIO rats chronically treated with OFS+MET exhibited a best increased glucose stabilizing ability compared with all the groups that received placebo, individual OFS, and MET. There is no significant difference between the MET-treated group and the OFS+MET-treated group in glucose lowering at weeks 4 and 8. However, there is a tendency for OFS+MET treatment to exert a slightly better glucose-stabilizing effect than individual treatment of MET.

**Figure 3:**
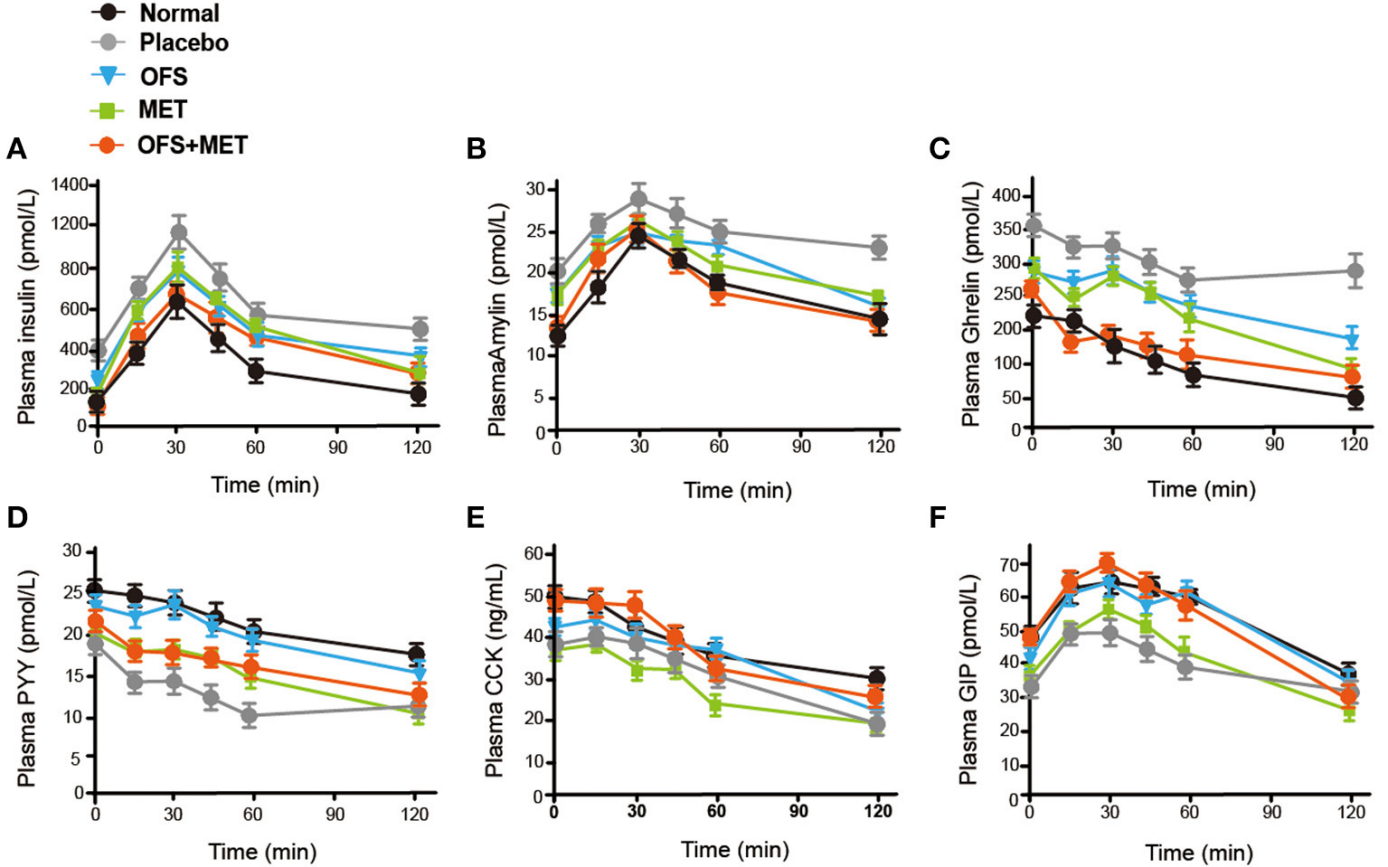
OFS, MET, and OFS+MET treatment alter the plasma gut hormones. **(A)** Insulin; **(B)** amylin; **(C)** ghrelin; **(D)** PYY; **(E)** CCK; **(F)** GIP. All data are expressed as mean ± S.E.M. (*n* = 8).

We further evaluated whether and how the plasma levels of appetite-related hormones were affected by the administration of OFS, MET, and OFS+MET during the OGTT at week 8. As shown in Figure 3 and Table 3, OFS+MET treatment significantly reduced the fasting concentration and AUC of insulin, amylin, and ghrelin (Figures 3B,C; Table 3), and increased the fasting levels and AUC of peptide YY (PYY), cholecystokinin (CCK), and GIP, which were close to the normal ones. These changes explained an obvious reduction of food intake and body weight due to appetite suppression lead (Figures 3D–F; Table 3).

**Table 3:** Area under the curve for the insulin and appetite-related hormones levels of OFS-, MET-, and OFS+MET-treated DIO rats during OGTT at week 8.

**Treatment**	**Control**	**OFS**	**MET**	**OFS+MET**
Insulin, nmol/L.min	60.5 ± 6.4	51.7 ± 6.6	45.3 ± 3.0^*^	42.8 ± 3.1^**^
Amylin, nmol/L.min	495.2 ± 19.1	489.5 ± 11.8	421.8 ± 15.4^*^	408.2 ± 12.9^**^
Ghrelin, nmol/L.min	25.4 ± 3.3	25.2 ± 3.1	23.9 ± 2.4	22.1 ± 1.7^*^
PYY, nmol/L.min	276.1 ± 22.7	259.1 ± 29.5	250.9 ± 19.1	248.6 ± 16.4^*^
CCK, nmol/L.min	95.1 ± 8.3	89.1 ± 8.2	92.2 ± 7.1	90.5 ± 7.0
GIP, nmol/L.min	5.2 ± 1.1	195.1 ± 11.2	175.2 ± 8.8^**^	181.6 ± 10.3

Values represent the mean ± S.E.M. (n = 8).

* P < 0.05,

** P < 0.01,

*** *P < 0.001 compared to saline-treated control*.

### Comparisons of Differential Profiles in Gut Microbiota Among OFS-, MET-, and OFS+MET-Treated DIO Rats

To explore whether the 8-week treatments of OFS, MET, and OFS+MET affect the gut microbial composition of DIO rats or not, we detected 40 fecal samples from each group using the 16S rRNA gene sequencing-based method. As a result, the 16S rRNA sequencing yielded probably 83,000 clean reads (duplicate reads > 95%) in each sample after a strict process including quality control, denoising, and the removal of chimera. Then, the clean reads were clustered into operational taxonomic units (OTUs) and then carefully assigned to taxa from phylum to species level.

**Figure 4:**
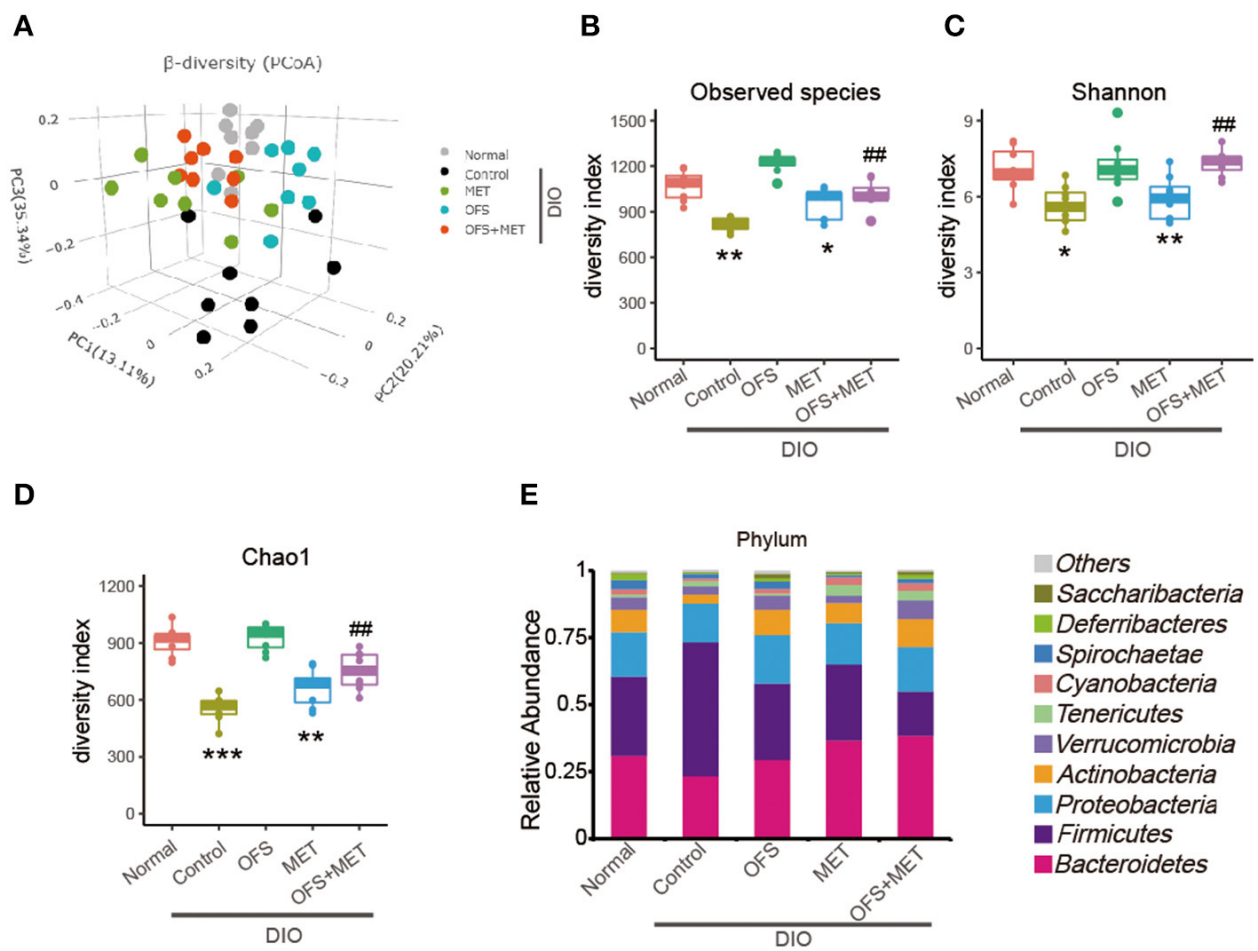
Differential profiles of gut microbiota among Control, MET, OFS, and MET+OFS. **(A)** 3D plot of unweighted UniFrac PCoA of intestinal microbiota extracted from rat fecal samples. Compared with the gut microbial diversity including **(B)** Observed species index; **(C)** Shannon index; **(D)** Chao1 index; **(E)** Phylogenetic profiles of gut microbes among Control, MET, OFS, and MET+OFS at the phylum level. Data are shown as mean ± S.E.M. (*n* = 8) (*P < 0.05, **P < 0.01, #Tukey test).

Beta diversity analysis was performed by principal coordinates analysis (PCoA) of the unweighted UniFrac distances of gut microbiota samples to explore the similarity of bacterial community patterns among all the DIO rat groups. As a result, a clear separation of the microbial community is shown in the placebo-treated control group, while the OFS and OFS+MET group tended to be closer to the normal group as compared with the MET group (Figure 4A). Alpha diversity is the mean diversity of bacteria or species within a community or habitat. Observed species', Shannon's, and Chao1's diversity indexes were also used to evaluate the community diversity of gut microbiota among all the treatment groups. Compared with the normal group, a significant decrease was observed in all the three diversity indexes among the control and Met group, while the OFS treatment exerts a non-obvious increase in Observed species and Chao1's diversity. Interestingly, the combination of OFS and MET could reverse the downward effect from MET on all three indexes, indicating that the supplement of OFS helps to reverse the decrease in gut microbiome diversity induced by MET administration (Figures 4B–D).

As shown in Figure 4E, the gut microbiota of the DIO rats were mainly composed of *Bacteroidetes, Firmicutes, Proteobacteria, Actinobacteria*, and *Verrucomicrobia* at the phylum level, especially for *Bacteroidetes* and *Firmicutes*. Compared with the normal rats, abundance of *Firmicutes* in placebo-treated DIO rats was obviously increased, while the *Bacteriodetes* and *Actinobacteria* were decreased relatively. In addition, the ratio of *Bacteriodetes*/*Firmicutes*, a measure associated with obesity and increased host inflammation, was reduced in the control group. In contrast, OFS+MET treatment increased the abundance of *Bacteriodetes* and *Actinobacteria*, which not only partially rescue the declining ratio of *Bacteriodetes/Firmicutes*, but also substantially correct the loss in obesity, respectively. Notably, abundance of *Verrucomicrobia*, which was a kind of beneficial bacterium helping to improve insulin resistance and reduce weight, in the OFS group and OFS+MET group was similar to the normal ones, while these were significantly decreased in placebo-treated DIO rats.

**Figure 5:**
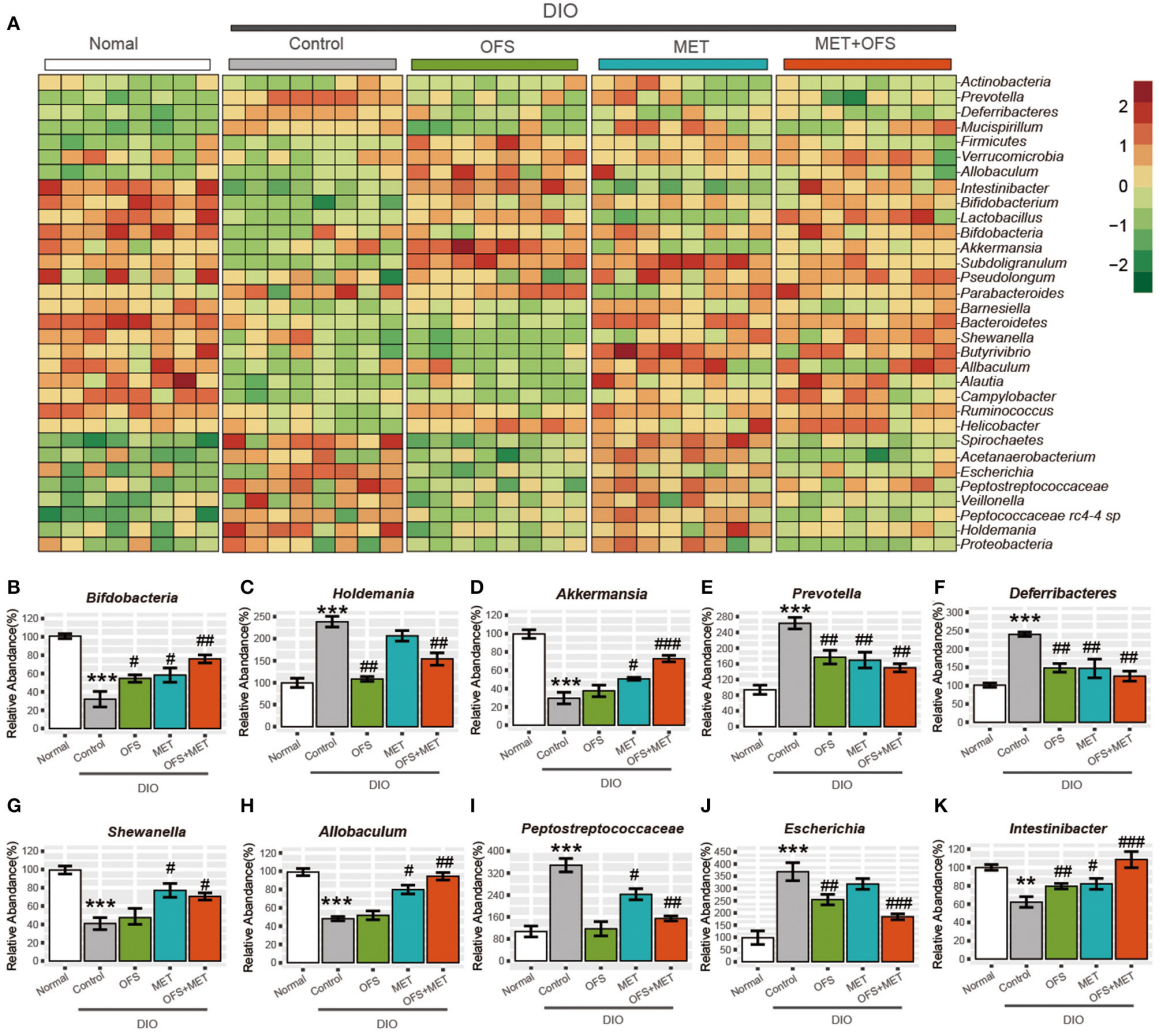
Effects of oligofructose and metformin on levels of gut microbiota in DIO rats. **(A)** Heat map was constructed comparing significantly different abundance of species. **(B–K)** The top10 significantly different microbial community at the species level (mean ± SEM, Student *t*-test, FDR, *n* = 8; **P < 0.01, ***P < 0.001, vs. Normal group; ^#^*P* < 0.05, ^*##*^*P* < 0.01, ^*###*^*P* < 0.001, vs. Control group).

Compared with abundance of *B*. *pseudolongum* in normal rats, obesity induced an obvious decline in this species, while the treatment of OFS and MET led to a reverse trend. In addition, combination treatment of OFS and MET in DIO rats was associated with a more significant improvement. A similar pattern is shown in the abundance of *Bifdobacterium* species (Figure 5A). Since *B*. *pseudolongum* and *Bifdobacterium* are reportedly related to positive health effects, including improved gut barrier integrity, stimulated immune response, and reduced inflammation caused by diet-induced obesity, the abovementioned shifts indicate the potentially enhanced protection from OFS+MET against obesity-induced intestinal and systemic effects, compared with the treatment of individual OFS or MET only (29, 30). In addition, MET treatment induced increase in the abundance of *Shewanella* and *Allobaculum* associated (Figures 5G,H) with improved metabolic endotoxemia, while there are no obvious changes in the OFS group. Interestingly, both of the microbes were obviously increased with OFS+MET treatment.

Interestingly, two members of the *Peptostreptococca* species (*Peptococcaceae rc4-4* sp. and *Peptostreptococcaceae* sp.), which were associated with intestinal inflammation and obesity, were both significantly reduced under OFS or OFS+MET treatment (Figure 5I). However, treatment of individual MET increased and maintained the abundance of *Peptococcaceae rc4-4* sp. and *Peptostreptococcaceae* sp., respectively, indicating that OFS may be an effective complement to MET on the improvement of inflammation and obesity reduction (29, 31). Similar improvement of OFS on the function of MET was also shown in the alternation of the abundance of microbes related with side effects of MET treatment. Both on a compositional and functional level, we that found significant microbiome, including enrichment of virulence factors and gas metabolism genes, could be attributed to the significantly increased abundance of *Escherichia* species (Figure 5J), which was obviously reduced by the treatment of OFS (32–34).

Not only that, *Intestinibacter* was previously reported to improve the resistance to oxidative stress, be able to degrade fucose, and possess the genetic potential for sulfite reduction, including part of an assimilatory sulfate reduction pathway, and, in this research, has been shown to obviously increase in abundance under OFS treatment (33, 34). Furthermore, the addition of OFS also reversed the down-regulatory effect on the abundance of *Intestinibacter* under individual MET treatment (Figure 5K).

Our results suggest partial gut microbial mediation of both therapeutic and adverse effects of the OFS and MET, especially since the addition of OFS effectively improved the side effects related to microbes under MET treatment, although further *in vivo* validation is required to conclude causality and to verify our finding on the whole animal level.

### Effects of Fecal Microbiota Transplantation on Antibiotics-Treated DIO Rats

**Figure 6:**
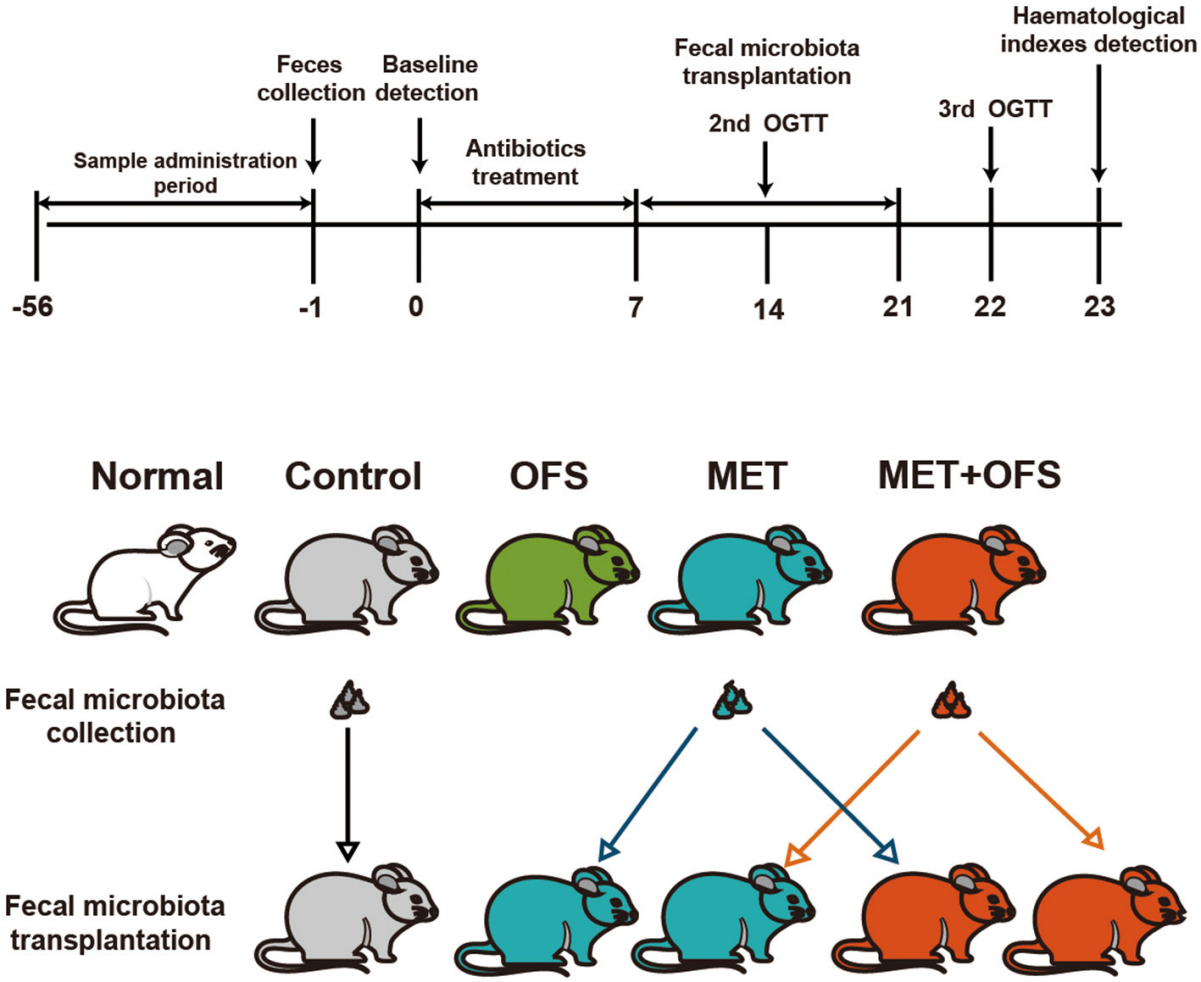
Schedule of fecal microbiota transplantation in antibiotics-treated DIO rats among the placebo, MET, and OFS+MET group.

To investigate whether the OFS+MET-altered microbiota could effectively contribute to the improvement of side effects and other metabolic profiles of MET, we exchanged and transferred fecal samples from each group to antibiotics-induced germ-free rats, and the design of this experiment is shown in Figure 6.

**Figure 7:**
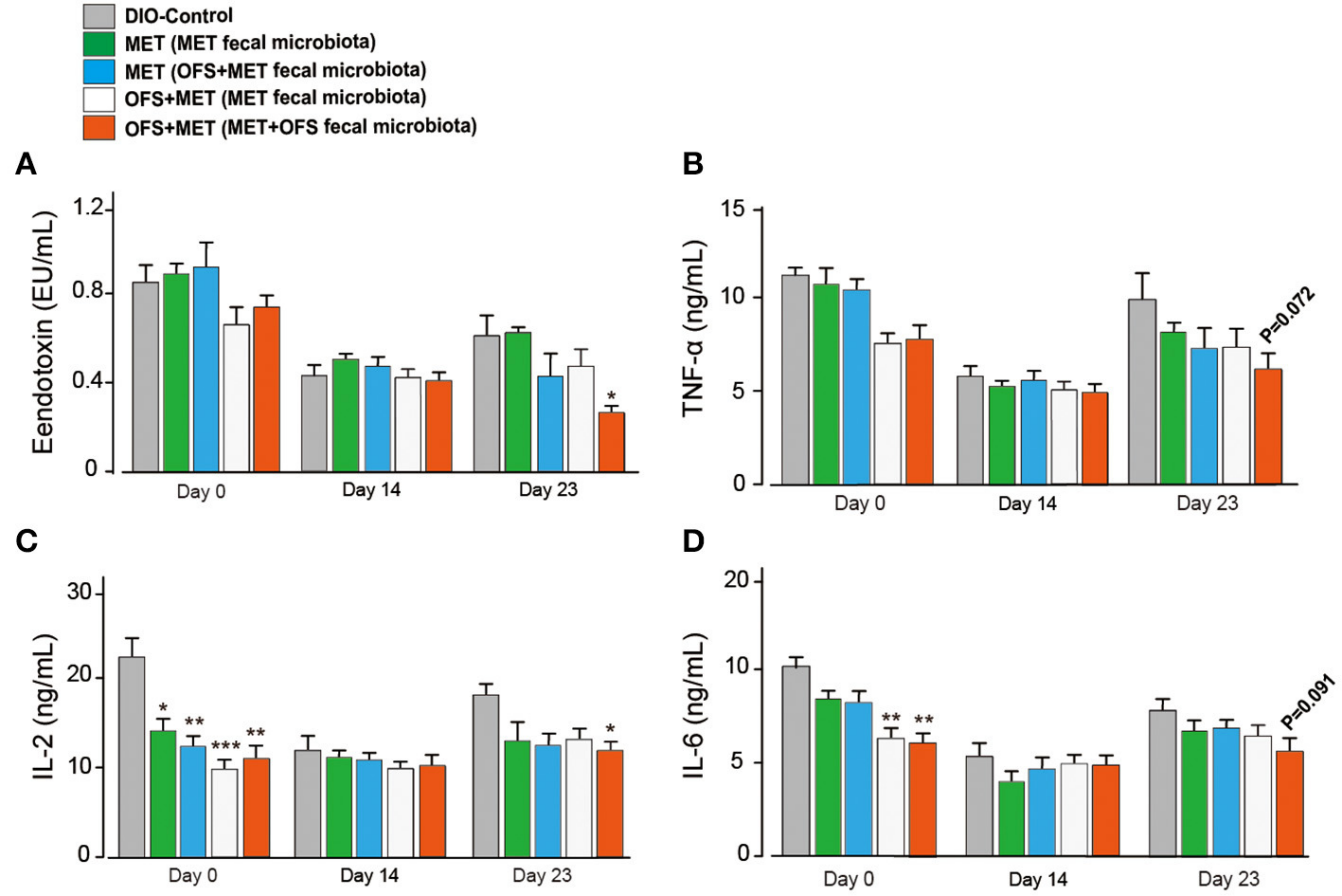
Translation of fecal microbiota from the MET- and OFS+MET-treated group alters the plasma inflammation-associated indexes. **(A)** Endotoxin; **(B)** TNF-α; **(C)** IL-2; **(D)** IL-6. All data are expressed as mean ± S.E.M. (*n* = 4). *P* < 0.05, 0.01 and 0.001 using one-way ANOVA vs. DIO control group (*, **, ***).

**Figure 8:**
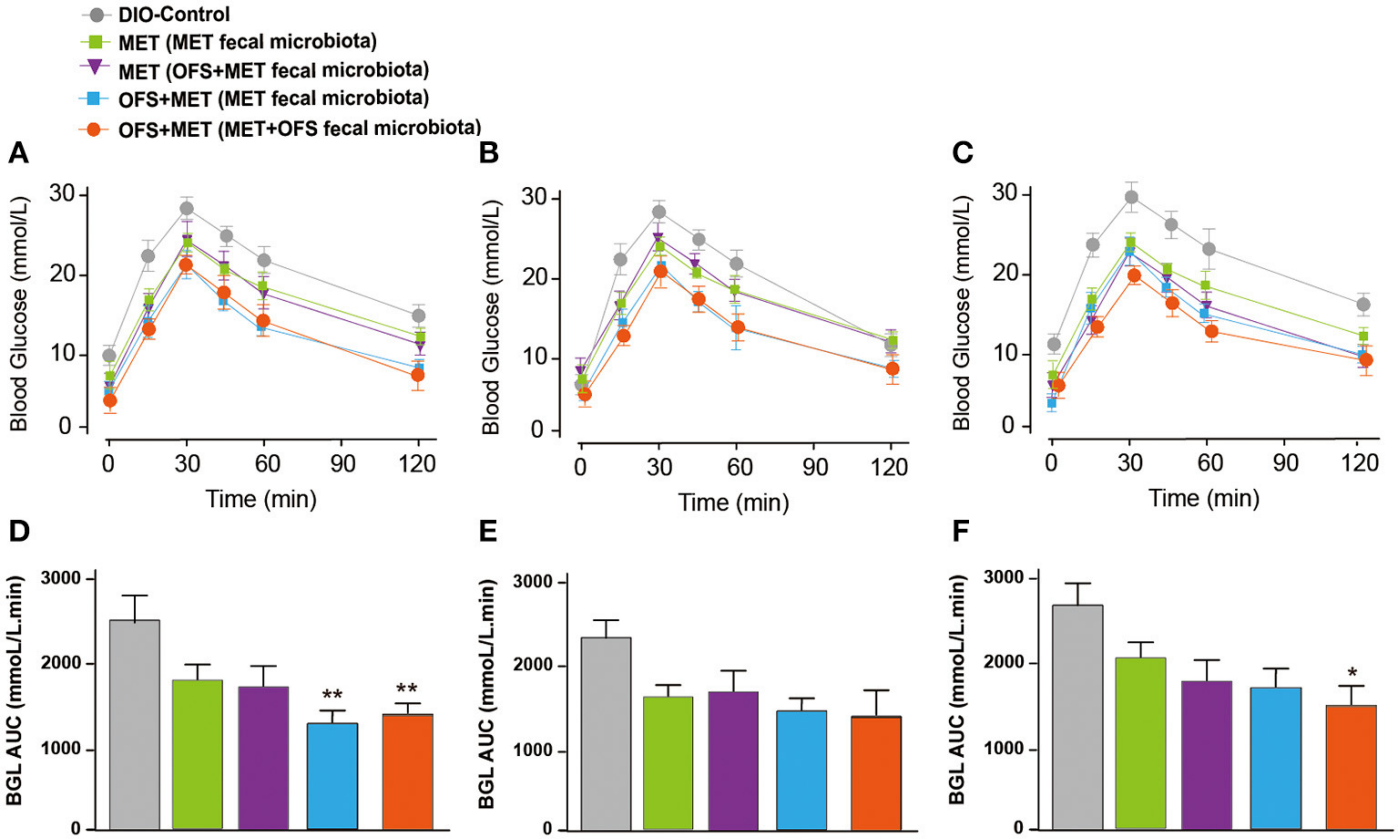
Effects of translation of fecal microbiota from the MET- and OFS+MET-treated group on glucose tolerance of each group. OGTT was conducted at **(A,D)** 1 day before antibiotics treatment, **(B,E)** Day 12, **(C,F)** Day 33. *P* < 0.05, 0.01 using one-way ANOVA vs. DIO control group (*, **). All data are expressed as mean ± S.E.M. (*n* = 4).

The serum levels change of endotoxin, TNF-α, IL-2, and IL-6 are shown in Figure 7. The MET group (transplantation of MET fecal microbiota, green histogram in Figure 7) showed a continuously deteriorating trend compared with the MET group (transplantation of OFS+MET fecal microbiota, blue histogram in Figure 7). The opposite trend is shown in two OFS+MET groups (transplantation of MET or OFS+MET fecal microbiota, white or red histogram, respectively, in Figure 7), indicating that transplantation of OFS+MET fecal microbiota has positive effects on inflammation-related factors that were reportedly induced by the administration of MET (21, 32). We further investigate the effects of fecal microbiota transplantation on glucose tolerance of DIO rats. Results are shown in Figure 8; fecal transfer to germ-free rats resulted in improved glucose tolerance in recipients of MET+OFS-altered microbiota, with overall substantial improvement of glucose metabolism, thus indicating that MET+OFS-adapted microbiota could contribute to more beneficial effects on glucose homeostasis compared with individual treatment of MET.

### Effects of Fecal Microbiota Transplantation on Glucose Tolerance and Metabolic Profiles in Germ-Free DIO Mice

**Figure 9:**
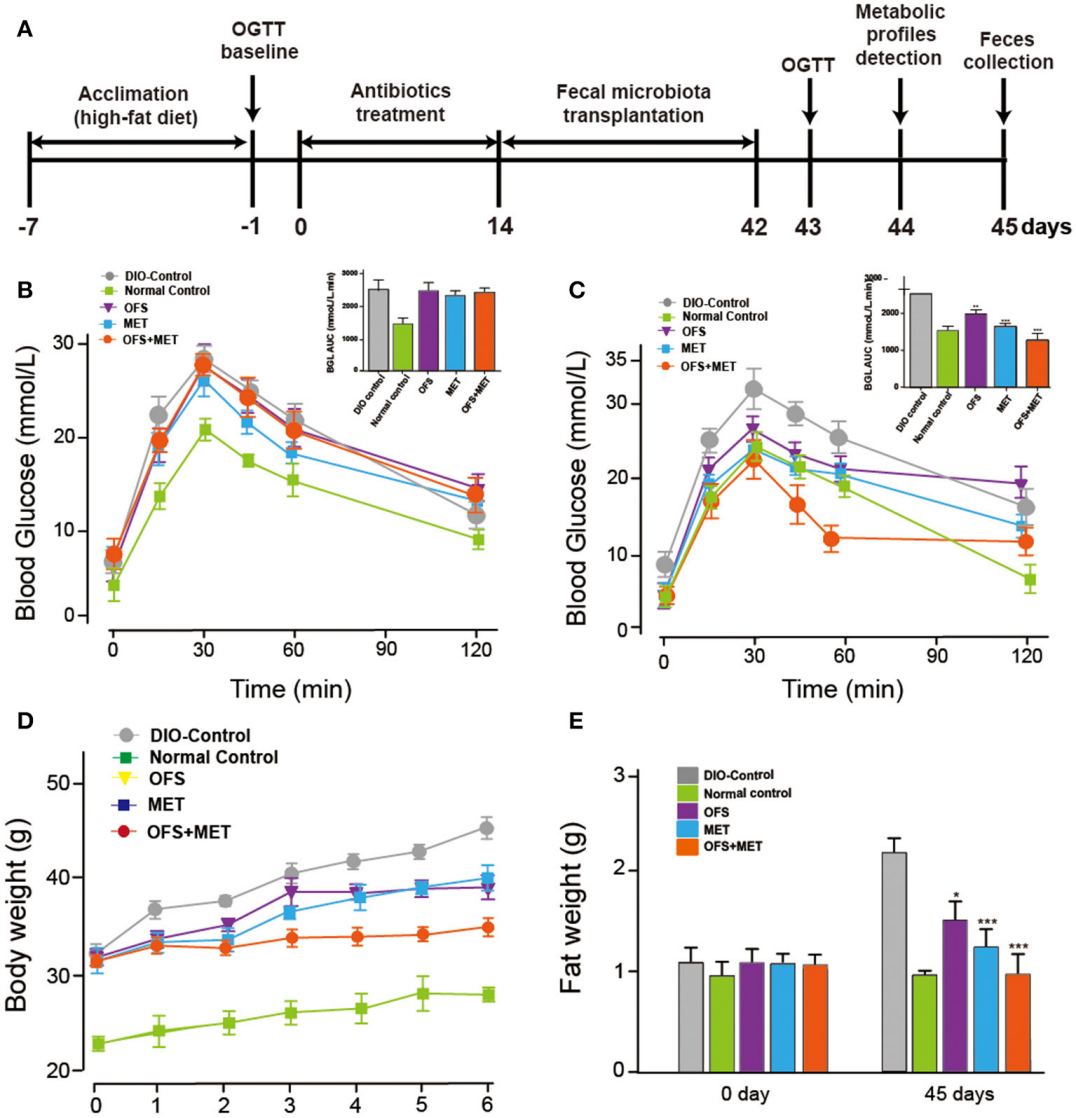
Effects of OFS, MET, and OFS+MET-altered fecal microbiota transplantation on germ-free DIO mice. **(A)** Schedule of fecal microbiota transplantation in pseudo-germ-free mice; **(B)** OGTT baseline; **(C)** OGTT after the fecal microbiota transplantation; **(D)** body weight, and **(E)** fat weight. *P* < 0.05, 0.01, 0.001 using one-way ANOVA vs. DIO-control group (*, **, ***). All data are expressed as mean ± S.E.M. (*n* = 8).

After intake of antibiotics through oral administration for 2 weeks, fecal microbiota from rats treated with placebo, OFS, MET, or MET+OFS were transplanted into pseudo-germ-free mice for 4 consecutive weeks (Figure 9A). The 4- to 6-week-old germ-free DIO mice were fed a 60% high-fat diet during the entire experiment for 45 days. After the 7-day antibiotics treatment, all the mice, with similar body weight and fasting blood glucose levels, were divided into four groups and then received an OGTT as baseline (Figure 9B). Then, the next OGTT were conducted at day 43, and results are presented in Figure 9C, indicating that the transplantation of fecal microbiota from OFS+MET significantly improved the glucose tolerance of the mice compared with the DIO control. Significant differences were also observed in body weight and fat mass between the mice that received placebo and OFS+MET-altered microbiota (Figures 9D,E).

Not only that, we also found that OFS+MET-altered fecal microbiota transplantation significantly decreased mesenteric and epididymal fat and total fat coefficient compared with the DIO control group (Table 4). In addition, especially for the results, Lee's index, an effective parameter to evaluate the obesity in adult rats, in the OFS+MET group was obviously improved compared to those in the DIO control group, which had a slightly better effect than the normal group.

**Table 4:** Comparison of visceral mass, total fat coefficient, and Lee's index among the OFS, MET, and OFS+MET-altered fecal microbiota transplantation groups.

**Groups**	**Visceral mass (g)**			**Total fat coefficient (%)**	**Lee's index**
	**Mesenteric fat**	**Epididymal fat**	**Perirenal fat**		
DIO control	0.47 ± 0.11	0.52 ± 0.14	0.31 ± 0.15	0.51 ± 0.09	31.32 ± 3.42
Normal control	0.41 ± 0.02	0.31 ± 0.07	0.14 ± 0.05	0.34 ± 0.06	26.11 ± 1.73
OFS	0.21 ± 0.01***	0.39 ± 0.05	0.31 ± 0.11	0.43 ± 0.07	30.25 ± 1.88
MET	0.24 ± 0.02***	0.43 ± 0.08	0.29 ± 0.15	0.36 ± 0.08	29.91 ± 3.12
OFS+MET	0.16 ± 0.01***	0.35 ± 0.03*	0.28 ± 0.13	0.31 ± 0.02**	25.17 ± 1.92**

P < 0.05, 0.01, 0.001 using one-way ANOVA vs. DIO control group (*, **, ***). All data are expressed as mean ± S.E.M. (n = 8).

**Figure 10:**
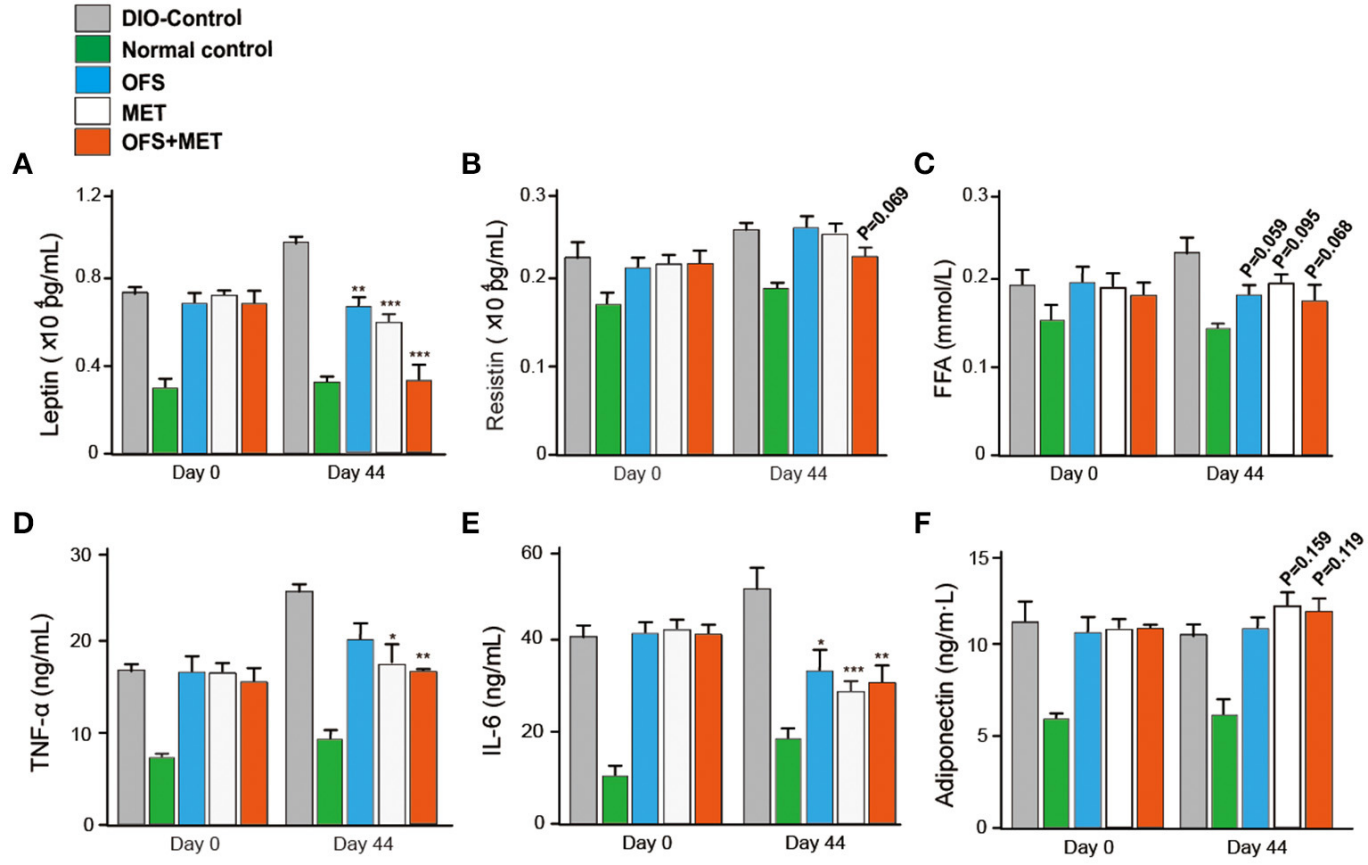
Effects of OFS, MET, and OFS+MET-altered fecal microbiota transplantation on obesity-related disease risk factors. The measurement of leptin **(A)**, resistin **(B)**, FFA **(C)**, TNF-α **(D)**, IL-6 **(E)**, and adiponectin **(F)** levels in DIO mice. *P* < 0.05, 0.02, 0.001 using one-way ANOVA vs. DIO-control group (*, **, ***). All data are expressed as mean ± S.E.M. (*n* = 8).

We further evaluated whether the blood biochemical indexes were also affected by the fecal microbiota transplantation at day 44. As shown in Table 5, transplantation of fecal microbiota obtained from the OFS+MET group exerted a down trending in the CHOL level and significantly reduced levels of ALT, TG, and LDL-C levels compared with the DIO control. As shown in Figure 10, the serum levels of resistin, free fatty acid (FFA), and adiponectin were not significantly changed but exerted a more obvious downtrend in the germ-free mice that received fecal microbiota from OFS+MET-treated rats, whereas leptin and several inflammation-related factors, including TNF-α and IL-6, were all relatively declined due to the fecal transfer of MET+OFS-altered microbiota to germ-free mice.

**Table 5:** Comparison of TG, CHOL, HDL-C, and LDL-C levels among the OFS, MET, and OFS+MET-altered fecal microbiota transplantation groups.

**Variable**	**ALT (U/L)**	**TG (mmol/L)**	**CHOL (mmol/L)**	**HDL-C (mmol/L)**	**LDL-C (mmol/L)**
Normal control	43.32 ± 7.13	0.75 ± 0.05	2.82 ± 0.18	1.71 ± 0.28	0.17 ± 0.02
DIO control	31.71 ± 9.52	0.87 ± 0.08	1.92 ± 0.08	2.11 ± 0.31	0.54 ± 0.03
OFS	40.12 ± 7.13	0.97 ± 0.12	2.13 ± 0.21	1.51 ± 0.34	0.61 ± 0.05
MET	38.80 ± 10.17	0.80 ± 0.07	2.41 ± 0.28	2.01 ± 0.11	0.45 ± 0.08*
OFS+MET	36.64 ± 8.12*	0.66 ± 0.09*	2.45 ± 0.32	2.15 ± 0.37	0.31 ± 0.11*

ALT, alanine transaminase; CRP, C-reactive protein; TG, triglyceride; CHOL, total cholesterol; HDL-C, high-density lipoprotein cholesterol; LDL-C, low-density lipoprotein cholesterol. P < 0.05, 0.02, 0.001 using one-way ANOVA vs. DIO control group (*, **, ***). All data are expressed as mean ± S.E.M. (n = 8).

## Discussion

T2DM and obesity, as the most impactful chronic diseases, have resulted in an increasing public medical expenditure and fatality rate worldwide. Obese individuals are usually accompanied by many serious complications, such as type 2 diabetes mellitus (T2DM), hypertension, and hyperlipidemia. The development of chronic diseases is usually affected by many factors including genetic, physiological, and behavioral factors (1). Due to the high pathogenesis complexity, significant efforts are being conducted to discover new strategies in the prevention and treatment of T2DM and overweight. MET, an oral antidiabetic agent, is widely utilized in the treatment of T2DM. OFS, as a commonly used prebiotic and non-digestible fermentable dietary fiber, exerts an antidiabetic effect, reducing energy intake and fat mass. Accumulating studies implicate that both the MET- and OFS-altered gut microbiota may play roles in the improvement of T2DM and obesity (7–11, 27, 33). Combination of MET and OFS displayed a novel and potential approach to treat T2DM and obesity. The present work aims to observe if the combination of MET and OFS enhanced the individual effects of these treatments. Based on evidences of MET- or OFS-altered gut microbiota mediating some antidiabetic and anti-obesity effects, we hypothesized that potentiated effects on improved glycemia and weight reduction would be determined.

In this study, we investigate the combination effects of OFS (10% wt/wt) and MET (150 mg/kg) on the metabolism of animals using rats with diet-induced obesity for 8 consecutive weeks. Results showed that OFS+MET treatment, but not TG and CHOL decrease, had obvious improvement on fat weight, %fat/body and %HbA1c (Figure 1A), weight reduction, food intake, and energy intake (Table 1). In addition, significantly decreased %HbA1c, fasting blood glucose, insulin level, and HOMA-IR in DIO rats are also listed in Table 2 in parallel with the improved glucose tolerance (Figure 2) after 8 weeks of treatment compared with individual treatment. According to previous research, reduced food and energy intake were closely related to the enhanced secretion of satiety hormones, such as GIP and PYY (35). In support of this purported mechanism, we observed that OFS+MET treatment significantly reduced the fasting concentration and AUC of insulin, amylin, and ghrelin (Figures 3B,C; Table 3), and increased the fasting levels and AUC of PYY, CCK, and GIP (Figures 3D–F; Table 3). Furthermore, we will explore whether the reduced food intake drives the phenotype via setting the pair-fed experiments in the follow-up studies.

To determine whether and how the combination of MET+OFS affects the gut microbiome, we performed 16s rRNA analysis of the obtained 40 fecal samples and further analysis of Beta and Alpha diversity. As a result, microbial community was clearly separated in the placebo-treated control group, while the OFS and OFS+MET group tended to be closer to the normal group as compared with the MET group (Figure 4A). Compared with the normal group, a significant decrease is observed in Species', Shannon's, and Chao1's diversity indexes among the control and MET group, while the OFS treatment exerts an obvious increase in Observed species and Chao1's diversity. Interestingly, combination of OFS and MET could reverse the downward effect from MET on the all three indexes related to the gut microbiome diversity of all treatment groups (Figures 4B–D).

As shown in Figure 5, OFS+MET treatment rescued the declining ratio of *Bacteriodetes/Firmicutes*, a measure associated with obesity and increased host inflammation, and substantially corrected the loss in obesity. Similarly, the abundance of *Verrucomicrobia* that helps to improve insulin resistance and reduce weight in the OFS+MET group was similar to the normal ones, while it decreased in the control group. In addition, obesity induced a significant decline of the abundance of *B*. *pseudolongum* and *Bifdobacterium*, which are reportedly related with positive health effects, such as decreasing inflammation, while the OFS+MET-treated group showed a more obvious reverse trend compared with individual treatments, indicating that the potentiated effects were indeed exist. Furthermore, the increased abundance of *Shewanella* and *Allobaculum*, which were associated with improved metabolic endotoxemia, are shown in the MET treatment group. Obvious changes in the OFS group were not observed yet, which indicated that MET treatment partially accounted for the effects of OFS on this beneficial bacterium. A similar pattern was shown in the abundance of *Peptococcaceae rc4-4* sp. and *Peptostreptococcaceae* sp. related to diet-induced obesity and intestinal inflammation. Both OFS and OFS+MET treatment significantly reduced these two bacteria. Interestingly, MET treatment increased and maintained the abundance of *Peptococcaceae rc4-4* sp. and *Peptostreptococcaceae* sp., respectively, indicating that OFS may be an effective complement to MET. In addition, *Intestinibacter* species was previously reported to improve the resistance to oxidative stress, be able to degrade fucose, and possess the genetic potential for sulfite reduction, including part of an assimilatory sulfate reduction pathway, and, in the present study, this species displayed an obvious increase in abundance under OFS treatment and the addition of OFS also reversed the down-regulatory effect on the abundance of *Intestinibacter* under only MET treatment. Furthermore, similar improvement of OFS on function of MET is also shown in the alteration of the abundance of *Escherichia* species, which was reportedly related to side effects of MET treatment including enrichment of virulence factors and gas metabolism genes. There is an increasing influence of gut microbiota on energy metabolism, and it will be critical to evaluate not only the effects of dietary factors known to alter gut microbiota but also the effects of pharmacological agents such as MET, OFS, or MET+OFS that are used to treat metabolic disease. It is likely that combination of MET- and OFS-altered gut microbiota might be associated with improved weight reduction, hyperglycemia, and insulin resistance. Not only that, some inflammation reactions and side effects induced by MET were also ameliorated due to the supplement of OFS.

In this study, the combination of MET and OFS was found to show potentiated effects on hyperglycemia, insulin resistance, and side effects related to gut microbiota compared with individual action. It is likely that MET+OFS-altered gut microbiota plays a role in these improvements. In order to further determine whether the OFS+MET-altered microbiota could effectively contribute to the improvement of side effects related to factors and other metabolic profiles induced by MET, the DIO rats treated with MET and MET+OFS were divided into two groups receiving fecal microbiota from the abovementioned two groups after the treatment of antibiotics for consecutive 12 days (Figure 6). Not surprisingly, the glucose tolerance and serum levels of endotoxin, FFA, TNF-α, IL-2, and IL-6 were all sustainably improved among OFS+MET-treated DIO rats (with OFS+MET fecal microbiota), while the MET-treated ones (with MET fecal microbiota) were shown in a reverse trend (Figures 7, 8).

Further, the transplantation of the fecal microbiota from DIO rats treated with OFS, MET, and OFS+MET, respectively, to antibiotics-induced germ-free mice showed that the OFS+MET-altered fecal microbiota could still improve glucose tolerance, weight reduction, Lee's index, and obesity-related disease risk factors (Figure 9). Interestingly, fecal sample transfer to the germ-free DIO mice resulted in an obviously improved glucose tolerance in the ones that received the MET+OFS-altered microbiota compared with the individual MET-adapted microbiota-treated ones, thus indicating that OFS supplementation to MET administration, in comparison to individual MET, provides more beneficial effects on glucose homeostasis and even contributes to the overall substantial improvement of glucose metabolism (Figures 9B,C).

In conclusion, our results suggest that the combination of MET and OFS induced gut microbial mediation of more perfect therapeutic effects, including improved glucose tolerance, HA1bc, weight reduction, and other blood biochemical indexes, compared with administration of individual MET or OFS. Based on previous reports (21, 32), significant microbiome alterations are consistent with well-known side effects of MET treatment, such as intestinal diseases and decreasing serum FFA. Most of these metformin-associated functional shifts, including enrichment of virulence factors and gas metabolism genes, could be attributed to the significantly increased abundance of *Escherichia* and other related species. In the present study, OFS is found to have potential to be a supplement for the most widely used antidiabetic treatment of MET, especially in terms of enhanced positive effects and improved negative effects. In addition, the potential molecular mechanism of these outcomes will be deeply explored in future research.

## Data Availability Statement

The datasets generated for this study can be found in NCBI accession number PRJNA596853 (http://www.ncbi.nlm.nih.gov/bioproject/596853).

## Ethics Statement

The animal study was reviewed and approved by The Animal Care Committee of Binzhou Medical University. Written informed consent was obtained from the owners for the participation of their animals in this study.

## Author Contributions

QL and RH: design and analysis of ideas. FZ: methodology. JZ: resources. SL: writing—original draft preparation. HL: writing—review and editing. QL: methodology and experimental operation.

### Conflict of Interest

The authors declare that the research was conducted in the absence of any commercial or financial relationships that could be construed as a potential conflict of interest.
